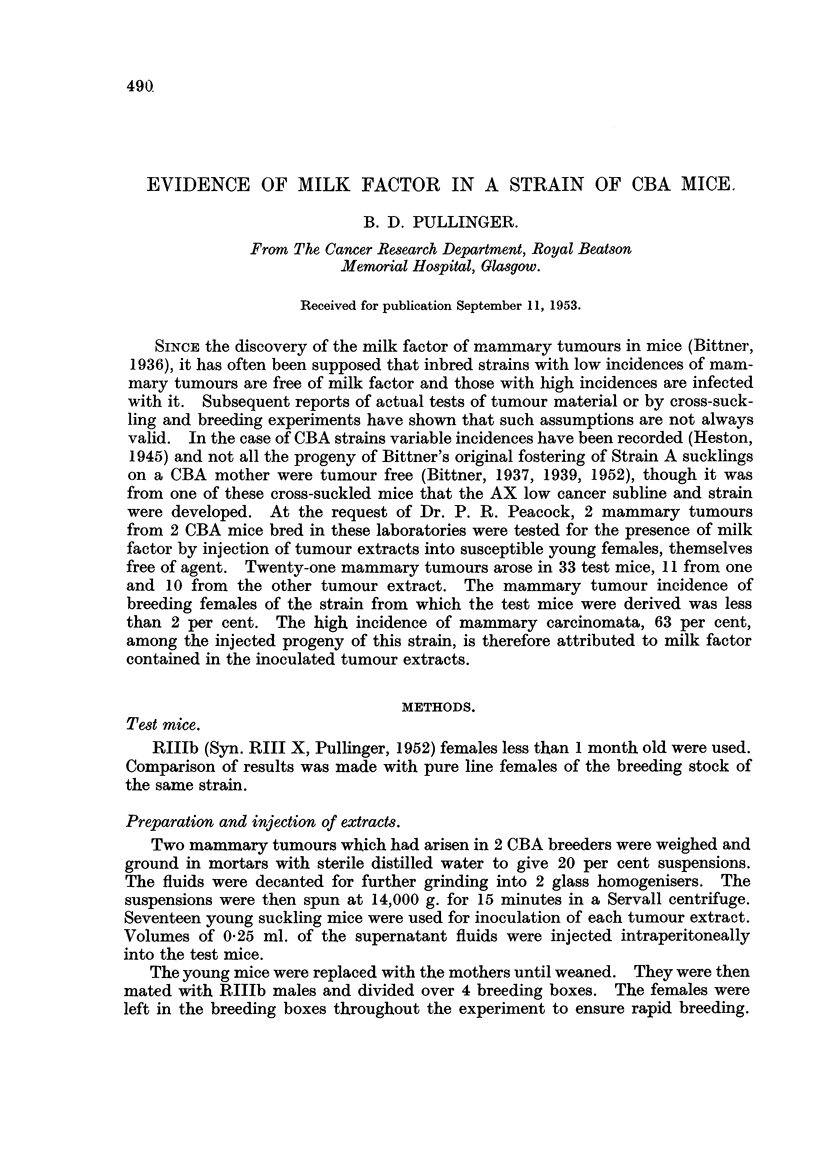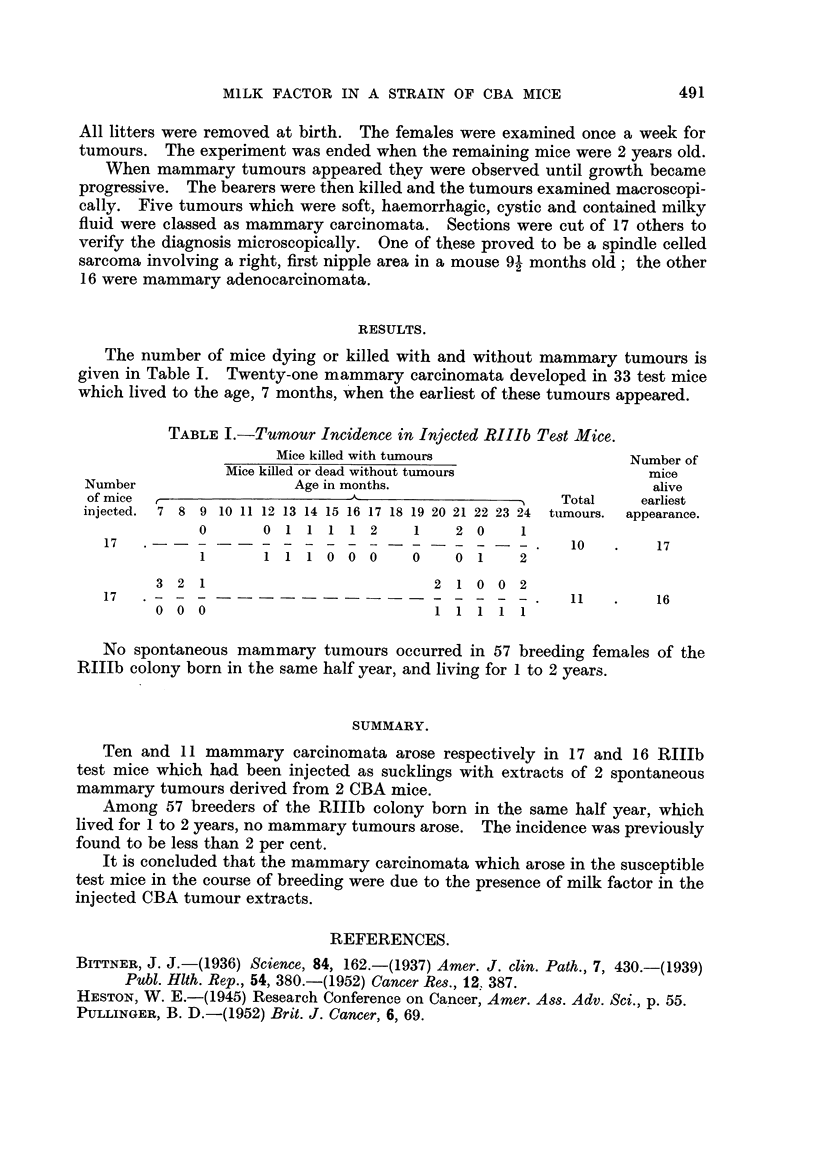# Evidence of Milk Factor in a Strain of CBA Mice

**DOI:** 10.1038/bjc.1953.51

**Published:** 1953-12

**Authors:** B. D. Pullinger


### EVIDENCE OF MILK FACTOR IN A STRAIN OF CBA MICE.

B. D. PULLINGER.

*From The Cancer Research Department, Royal Beatson Memorial Hospital, Glasgow.*

Received for publication September 11, 1953.

SINCE the discovery of the milk factor of mammary tumours in mice (Bittner, 1936), it has often been supposed that inbred strains with low incidences of mammary tumours are free of milk factor and those with high incidences are infected with it. Subsequent reports of actual tests of tumour material or by cross-suckling and breeding experiments have shown that such assumptions are not always valid. In the case of CBA strains variable incidences have been recorded (Heston, 1945) and not all the progeny of Bittner's original fostering of Strain A sucklings on a CBA mother were tumour free (Bittner, 1937, 1939, 1952), though it was from one of these cross-suckled mice that the AX low cancer subline and strain were developed. At the request of Dr. P. R. Peacock, 2 mammary tumours from 2 CBA mice bred in these laboratories were tested for the presence of milk factor by injection of tumour extracts into susceptible young females, themselves free of agent. Twenty-one mammary tumours arose in 33 test mice, 11 from one and 10 from the other tumour extract. The mammary tumour incidence of breeding females of the strain from which the test mice were derived was less than 2 per cent. The high incidence of mammary carcinomata, 63 per cent, among the injected progeny of this strain, is therefore attributed to milk factor contained in the inoculated tumour extracts.

### METHODS.

### *Test mice.*

RIIIb (Syn. RIII X, Pullinger, 1952) females less than 1 month old were used. Comparison of results was made with pure line females of the breeding stock of the same strain.

### *Preparation and injection of extracts.*

Two mammary tumours which had arisen in 2 CBA breeders were weighed and ground in mortars with sterile distilled water to give 20 per cent suspensions. The fluids were decanted for further grinding into 2 glass homogenisers. The suspensions were then spun at 14,000 g. for 15 minutes in a Servall centrifuge. Seventeen young suckling mice were used for inoculation of each tumour extract. Volumes of 0·25 ml. of the supernatant fluids were injected intraperitoneally into the test mice.

The young mice were replaced with the mothers until weaned. They were then mated with RIIIb males and divided over 4 breeding boxes. The females were left in the breeding boxes throughout the experiment to ensure rapid breeding.

All litters were removed at birth. The females were examined once a week for tumours. The experiment was ended when the remaining mice were 2 years old.

When mammary tumours appeared they were observed until growth became progressive. The bearers were then killed and the tumours examined macroscopically. Five tumours which were soft, haemorrhagic, cystic and contained milky fluid were classed as mammary carcinomata. Sections were cut of 17 others to verify the diagnosis microscopically. One of these proved to be a spindle celled sarcoma involving a right, first nipple area in a mouse 9½ months old ; the other 16 were mammary adenocarcinomata.

### RESULTS.

The number of mice dying or killed with and without mammary tumours is given in Table I. Twenty-one mammary carcinomata developed in 33 test mice which lived to the age, 7 months, when the earliest of these tumours appeared.

TABLE I.—*Tumour Incidence in Injected RIIIb Test Mice.*

Upper figure: Mice killed with tumours; lower figure: Mice killed or dead without tumours. Age in months.

| Number of mice injected. | | 7 | 8 | 9 | 10 | 11 | 12 | 13 | 14 | 15 | 16 | 17 | 18 | 19 | 20 | 21 | 22 | 23 | 24 | Total tumours. | Number of mice alive earliest appearance. |
|---|---|---|---|---|---|---|---|---|---|---|---|---|---|---|---|---|---|---|---|---|---|
| 17 | Mice killed with tumours | | | 0 | | | 0 | 1 | 1 | 1 | 1 | 2 | | 1 | | 2 | 0 | | 1 | 10 | 17 |
| | Mice killed or dead without tumours | | | 1 | | | 1 | 1 | 1 | 0 | 0 | 0 | | 0 | | 0 | 1 | | 2 | | |
| 17 | Mice killed with tumours | 3 | 2 | 1 | | | | | | | | | | | 2 | 1 | 0 | 0 | 2 | 11 | 16 |
| | Mice killed or dead without tumours | 0 | 0 | 0 | | | | | | | | | | | 1 | 1 | 1 | 1 | 1 | | |

No spontaneous mammary tumours occurred in 57 breeding females of the RIIIb colony born in the same half year, and living for 1 to 2 years.

### SUMMARY.

Ten and 11 mammary carcinomata arose respectively in 17 and 16 RIIIb test mice which had been injected as sucklings with extracts of 2 spontaneous mammary tumours derived from 2 CBA mice.

Among 57 breeders of the RIIIb colony born in the same half year, which lived for 1 to 2 years, no mammary tumours arose. The incidence was previously found to be less than 2 per cent.

It is concluded that the mammary carcinomata which arose in the susceptible test mice in the course of breeding were due to the presence of milk factor in the injected CBA tumour extracts.

### REFERENCES.

BITTNER, J. J.—(1936) *Science*, **84**, 162.—(1937) *Amer. J. clin. Path.*, **7**, 430.—(1939) *Publ. Hlth. Rep.*, **54**, 380.—(1952) *Cancer Res.*, **12**, 387.

HESTON, W. E.—(1945) Research Conference on Cancer, *Amer. Ass. Adv. Sci.*, p. 55.

PULLINGER, B. D.—(1952) *Brit. J. Cancer*, **6**, 69.